# Effects of Residential Environment and Lifestyle on Atopic Eczema Among Preschool Children in Shenzhen, China

**DOI:** 10.3389/fpubh.2022.844832

**Published:** 2022-05-16

**Authors:** Yanlin Liu, Shujie Sun, Duo Zhang, Wenchen Li, Zhenya Duan, Shaoyou Lu

**Affiliations:** ^1^School of Transportation and Environment, Shenzhen Institute of Information Technology, Shenzhen, China; ^2^College of Electromechanical Engineering, Qingdao University of Science and Technology, Qingdao, China; ^3^School of Public Health (Shenzhen), Sun Yat-sen University, Shenzhen, China

**Keywords:** atopic eczema, children, dietary habits, residential/household environment, lifestyle

## Abstract

Eczema, one of the most prevalent inflammatory skin diseases among children, is potentially influenced by genetic, environmental and social factors. However, few studies have investigated the effect of residential environment and lifestyle on childhood eczema. Therefore, this study conducted a cross-sectional study based on 2,781 preschool children in Shenzhen, China, during 2015–2016. Logistic regression models were employed to analyze the associations between residential/household environment, lifestyle, dietary habits and eczema in children. The prevalence of eczema among children in Shenzhen was 24.6%. Significant associations (increased odds >50%, *P* < 0.05) were found between childhood eczema and the factors of using composite wood floors (adjusted OR = 1.777 for doctor-diagnosed eczema, 1.911 for eczema-like symptoms), living in a villa/townhouse (aOR = 3.102, 2.156), the presence of mold or damp stains in the child's room (aOR = 1.807, 2.279), and rarely cleaning the child's room (aOR = 1.513, 1.540). In addition, watching TV/playing computer games for more than one hour per day was significantly associated with eczema (aOR = 1.172, 1.174). Notably, we found that eating rice/pasta one to three times per week may elevate the risk of eczema-like symptoms (aOR = 1.343), which warrants further investigation. In addition, ambient air pollution, in the covariates, may also affect childhood eczema. Therefore, avoiding these adverse factors and creating a low-risk environment are crucial to prevent childhood eczema.

## Introduction

Eczema, also known as allergic dermatitis, is one of the most prevalent inflammatory skin diseases among children. It usually occurs as pruritus at the back of the knees and the front of the elbow. About 15–30% of children all over the world suffer from eczema (1, 2). Although eczema is not life-threatening, it can lead to skin damage and cause a heavy burden on the quality of life of patients and their families, as well as consuming medical resources (3, 4). Moreover, eczema is a precursor to asthma and allergic rhinitis, and preventive measures for eczema are also aimed at preventing these more severe conditions later in life. Therefore, it is necessary to explore the key pathogenic factors in order to establish more effective preventive and therapeutic strategies.

The incidence of eczema may be associated with genetic factors, ambient air exposure, and social and economic status (5–8). However, the rapid urbanization and industrialization in China have changed people's consumption patterns, lifestyles and living environments (9–11). Meanwhile, the prevalence of eczema in children has also increased rapidly (12). Studies have reported that eczema in children from specific regions may be associated with the residential (13–16) and household environment (17–19). However, studies related to building materials and house types are rather limited by regions. Besides, dietary habits such as vegetable and fruit intake (20–23) and lifestyle factors e.g., parental smoking (14, 24) may also increase odds of eczema. But current findings have partially been conflicting, which may be because there are many confounders in dietary and lifestyle factors. However, no studies have incorporated all of these potential factors into investigation, making it difficult to explore the key influencing factors.

Hence, this study systemically investigated the associations of dietary habits, residential/household environment, and lifestyle and childhood eczema in Shenzhen, China. To the best of our knowledge, we provided the first comprehensive data on these potential influencing factors of childhood eczema in a typically urbanizing and industrializing region.

## Materials and Methods

### Study Protocol

**Figure 1:**
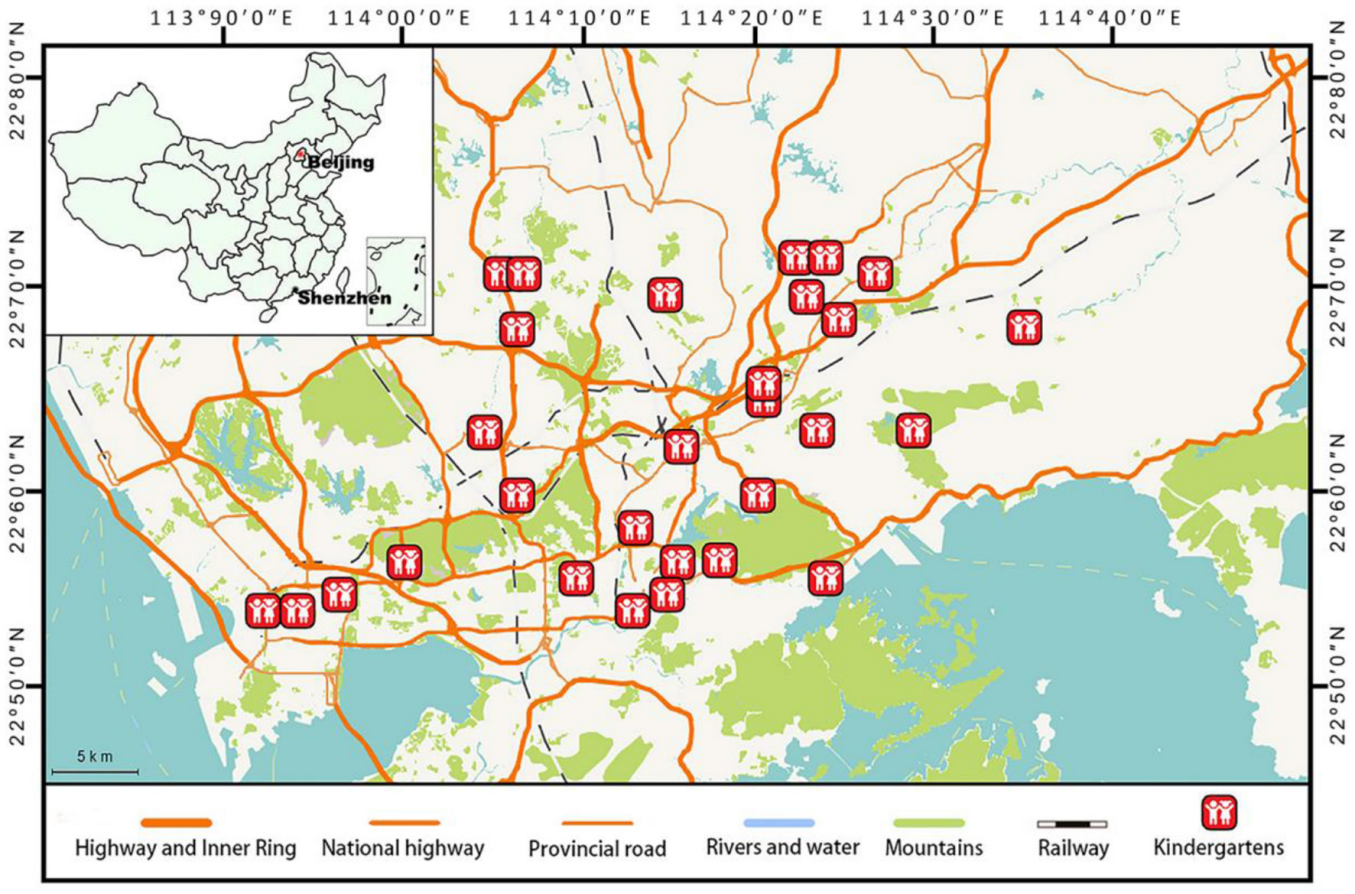
Distribution of the kindergartens among preschool children in this study *(n* = 2,781).

This study was based on a follow-up to the nationwide “China-Children-Homes-Health (CCHH)” study performed in 2010–2012 in multiple cities (12, 25). A total of 4,700 questionnaires were distributed to 30 kindergartens to investigate allergies and infections among children during 2015–2016 in Shenzhen, China (Figure 1). Shenzhen, which neighbors Hong Kong, is one of the most developed and urbanized cities in China, with a population of more than 13 million. Shenzhen features a subtropical maritime climate with a high annual temperature (24°C) and relative humidity of about 70%. Notably, Shenzhen has the largest number of kindergarten children (>500,000) in China.

Children in kindergartens were randomly sampled to represent the general population of preschool children in Shenzhen. Our questionnaire was based on the International Study of Asthma and Allergies in Childhood (ISAAC) (26) and a Swedish questionnaire regarding dampness in buildings and health (DBH) (27), with small changes to suit the conditions in the region. We collected information on eczema in childhood, residential/household environment, lifestyle and dietary habits of all family members. We entrusted the kindergartens to provide the questionnaires to the children's parents. To improve reliability, questionnaires were recommended to be filled out by the children's parents and returned to the kindergartens within 1 week.

We retrieved 4,614 filled questionnaires in total, with a very high response rate of 98.2%. First, 136 invalid samples with a large number of missing data were excluded. We then excluded 1,212 children aged younger than 3 years or older than 6 years. In addition, 485 questionnaires were invalid because of incomplete information about sex, age and eczema outcomes. Finally, a total of 2,781 samples with paired questionnaires were included in this study.

### Exposure Assessment

The residential/household environment represented the outdoor and indoor living environment. The lifestyles included living and dietary habits in the last 12 months before the survey. Relatively long exposure period was set to reflect the actual scenario of sensitization, because (1) eczema is a long-term disease and children are prone to recurrent attacks once eczema is triggered, and (2) the potential influencing factors such as dietary intake and lifestyles are generally stable within a long period.

Meats, seafood, fruits, vegetables, beans/grains, rice/pasta, eggs, milk, and fast food are considered common dietary components in China and were classified according to the frequency of their intake. Zero points represented never or occasional intake, one point was less than four times per week, and two points was four times or more per week (28).

The residential environment included the location (residential area and located near a highway or not), size, age, and the type of dwelling. The household environment, being the indoor environment formed by individual habits, encompassed mold, damp stains, water damage, window condensation, and house decoration status (floor materials and repainting the child's room or not). Lifestyles were defined as the daily routine and living habits of children and their families, including the frequency of cleaning and ventilating and airing bedclothes, the time spent watching TV/playing with a computer, keeping pets at home, and smoking in the child's room. Children were defined as being “exposed” when their questionnaires answered “Yes” to the corresponding questions.

### Eczema Outcomes

“DDE” was defined by the following question: “Has your child ever been diagnosed with eczema by doctor(s)?”

“ELS” was defined by reviewing the children's eczema-like symptoms in the past 12 months. The symptoms were based on the diagnostic criteria, including redness, itching, rash, dryness, desquamation or blisters, and seborrhea.

Children were defined as having “DDE” or “ELS” when their questionnaires answered “Yes” to the corresponding questions.

### Confounding Covariates

According to previous studies (16, 21, 29–31), parental atopy, breast feeding and a child's age, sex, and birth season are the most general potential confounders for the associations of dietary habits, residential/household environment, and lifestyle with childhood eczema (Table 1). The information on confounding covariates was selected from the questionnaire. Birth season was classified according to the international standard season dates, and we defined parental atopy as at least one of the child's family members (siblings, parents, and/or grandparents) having had at least one of the following diseases: asthma, eczema, or rhinitis (8). These covariates were adjusted for in Model 2 and Model 3.

**Table 1:** Covariates and demographic information among children with eczema (*n* = 2,781).

**Items**	**Sample size, *n*** ** (%)**	**Doctor-diagnosed eczema** **(Total:** ***N*** **= 685, 24.6%)**		**Eczema-like symptoms** **(Total:** ***N*** **= 859, 30.9%)**	
		**Prevalence, n** ** (%)**	***P*** **-value**	**Prevalence, n** ** (%)**	***P*** **-value**
**Total**	**2,781 (100)**				
Sex			0.941		0.759
Boys	1,490 (53.6)	370 (24.8)		461 (30.9)	
Girls	1,291 (46.4)	315 (24.4)		398 (30.8)	
Age during the survey			**0.045**		0.138
3 years-old	451 (16.2)	131 (29.0)		152 (33.7)	
4 years-old	687 (24.7)	164 (23.9)		216 (31.4)	
5 years-old	855 (30.7)	214 (25.0)		269 (31.5)	
6 years-old	788 (28.3)	176 (22.3)		222 (28.2)	
Birth season			0.320		0.105
Spring	708 (25.5)	164 (23.2)		202 (28.5)	
Summer	732 (26.3)	187 (25.5)		229 (31.3)	
Autumn	626(22.5)	170 (27.2)		217 (34.7)	
Winter	715 (25.7)	164 (23.0)		211 (29.5)	
Parental atopy			**<0.001**		**<0.001**
Yes	382 (13.7)	156 (40.8)		176 (46.1)	
No	2,399 (86.3)	529 (22.1)		683 (28.5)	
Breast feeding			0.368		0.178
Yes	2,121 (76.3)	522 (24.6)		672 (31.7)	
No	659 (23.7)	153 (23.2)		187 (28.4)	

*Sum of the number is not 2,781 due to missing data*.

*The values p < 0.05 were in bold*.

Ambient air pollution has been reported to be associated with increased odds of childhood eczema (8). Three main ambient air pollutants (PM_10_, SO_2_, and NO_2_) were selected to be adjusted for in Model 3. The daily mean concentrations of each air pollutant during 2008–2016 for each participant were obtained from 12 municipal monitoring stations for ambient air pollutants. Personal exposure to outdoor PM_10_, SO_2_, and NO_2_ was calculated by the inverse distance weighted (IDW) method (25).

### Statistical Analysis

We used binary logistic regression models to evaluate the associations of childhood eczema with dietary habits, residential/household environment, and lifestyle by adjusting for potential confounding covariates. Pearson's chi-square test was used to compare the differences in childhood eczema prevalence. Associations in the regression analysis were calculated as the odds ratio (OR) with a 95% confidence interval (95% CI). A *P* < 0.05 was considered statistically significant. First, a single-variable model was adopted to evaluate the crude ORs of residential/household environment and lifestyle for childhood eczema (Model 1). Second, parental atopy, breast feeding and the child's age, sex and birth season were adjusted in Model 2. Third, the effects of ambient air pollutants were further adjusted (Model 3). All statistical analyses were performed using SPSS software (version 18.0, SPSS Inc, Chicago, IL, USA).

## Results and Discussion

**Figure 2:**
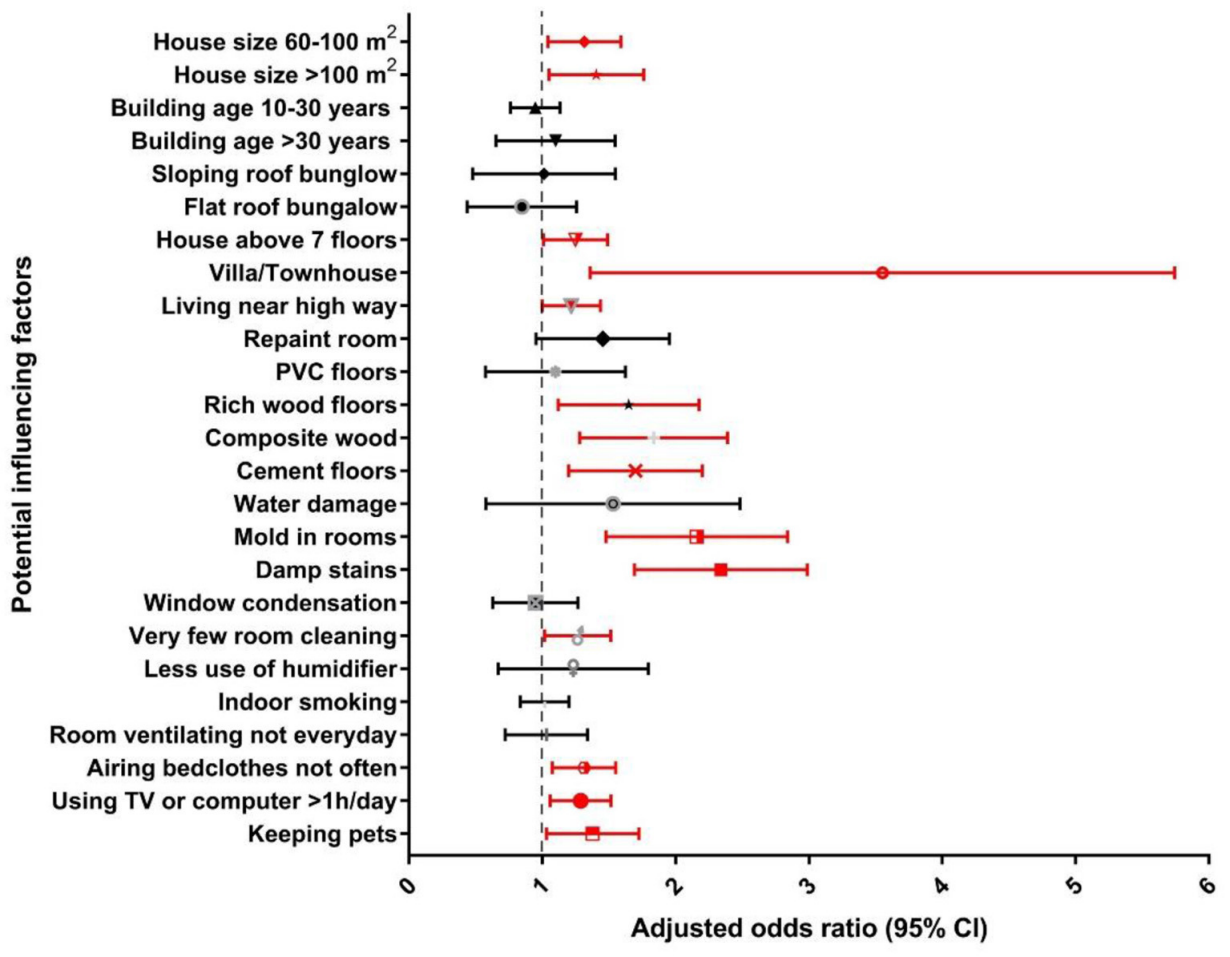
Associations between residential/household and lifestyle factors and diagnosed eczema among children in southern China.

This cross-sectional study is the first to systematically investigate the effects of living environment and lifestyle habits on childhood eczema in southern China. The effect of ambient air pollution was adjusted in the regression models. The results showed that diagnosed eczema among children was significantly associated with the residential/household environment, and lifestyles of their family members (Figure 2). However, we did not observe correlations between dietary factors and childhood DDE (*P* > 0.05), while fast food intake was found correlated with ELS (*P* < 0.05).

The prevalence of childhood eczema according to the covariates is shown in Table 1. Among the 2,781 respondents, 685 (24.6%) had doctor-diagnosed eczema, and 859 (30.9%) had experienced ELS in the past 12 months. Children with parental atopy (*P* < 0.001) had the highest prevalence of childhood eczema (40.8% for diagnosed eczema, 46.1% for ELS). An age difference was observed in DDE prevalence among children, with the highest prevalence among children aged 3 years (29.0%). Notably, we did not find an association between childhood eczema and sex or breast feeding. In this study, the prevalence of eczema in children was comparable to those in other Chinese cities including Wuhan (23%), Shanghai (21.1%), Changsha (28.6%), and Beijing (29.7%) (8, 16, 19), and to those in developed countries, such as Spain (21.9%), Sweden (23.0%), and the United States (20.0%) (32–35), suggesting that atopic eczema is a widely distributed problem of public health.

The associations of eczema prevalence in children and residential/household environment and lifestyle habits obtained by the chi-square test are shown in Table 2. The prevalence of DDE and ELS was relatively high with the use of rich wood, composite wood, or cement as floor materials, the behavior of rarely cleaning the child's room, and the presence of mold or damp stains in the child's room (*P* ≤ 0.001). Repainting the child's room, house size, building age, house type, airing bedclothes, time spent watching TV/playing with a computer, and keeping pets at home also had a significant impact on the prevalence of childhood eczema (*P* ≤ 0.01), especially living in a villa/townhouse (47.4%).

**Table 2:** Prevalence (%) of eczema or related symptoms stratified by residential/household environment and lifestyles (*n* = 2,781).

**Items**	**Doctor-diagnosed**		**Eczema-like**	
	**eczema**		**symptoms**	
	**Prevalence**,	***p*** **-**	**Prevalence**,	***p*** **-**
	**n (%)**	**value**	***n*** ** (%)**	**value**
Repaint in child's room	**0.047**			**0.009**
Yes	31.8		38.2	
No	24.3		30.6	
House size	**0.009**			**0.028**
<60 m^2^	21.4		28.5	
60–100 m^2^	25.9		31.3	
>100 m^2^	28.3		34.3	
Materials of floors in child's room	**< 0.001**			**< 0.001**
Stone / tile	22.5		28.4	
PVC	23.2		28.4	
Rich wood	31.4		35.7	
Composite wood	34.5		44.8	
Cement	32.6		38.2	
Others	19.2		26.6	
Living area	0.343			0.426
Urban	25.1		31.1	
Suburban	25.8		32.4	
Rural	18.6		24.5	
Others	24.8		31.4	
Building age	0.120			**0.002**
<10 years	25.9		31.4	
10–30 years	25.1		31.8	
> 30 years	26.2		33.8	
Don't know	19.8		25.8	
Living near high way	**0.046**			**0.036**
Yes	25.2		30.7	
No	24.4		30.8	
House type	**0.001**			**0.010**
Below 7 floors	23.4		29.6	
Sloping roof bungalow	22.7		29.3	
Villa/ Townhouse	47.4		47.4	
Flat roof bungalow	19.8		29.7	
Above 7 floors	27.5		33.6	
Frequency of humidifier use	0.774		0.539	
Everyday	23.3		32.0	
Several times a week	22.5		27.9	
Very few times	25.2		31.5	
Frequency of cleaning child's room	**0.001**		**< 0.001**	
Everyday	22.5		28.9	
Several times a week	28.8		35.0	
Very few times	23.9		32.4	
Water damage in child's room	0.264		0.520	
Yes	31.8		36.4	
No	24.4		30.8	
Smoking in child's room	0.741		0.960	
Yes	24.9		31.6	
No	25.8		31.2	
Mold in child's room	**< 0.001**		**< 0.001**	
Yes	40.2		45.1	
No	23.5		29.9	
Damp stains in child's room	**< 0.001**		**< 0.001**	
Yes	39.5		45.5	
No	23.0		29.3	
Window condensation in child's room	0.659		0.815	
Yes	23.3		29.8	
No	24.5		31.0	
Ventilating child's room	0.782		0.514	
Everyday	24.8		30.9	
Less than once a day	25.3		33.6	
Airing bedclothes	**0.002**		**0.021**	
Often	21.9		28.1	
Occasionally	27.8		34.3	
Never or rarely	18.3		26.7	
Time to watch TV or play computer	**0.002**	**0.023**		
<1 h per day	22.2		29.1	
> 1 h per day	27.2		33.1	
Pets keeping at home	**0.004**		**0.050**	
Yes	31.1		36.6	
No	24.1		30.3	

*The values p < 0.05 were in bold*.

Table 3 showed the associations between current dietary habits and eczema prevalence in children. Children with higher frequencies of eating rice/pasta every week had a significantly increased prevalence of DDE and ELS (*P* < 0.05). In addition, a relatively high prevalence of both DDE and ELS was found among children who never eat fruits or vegetables, have a moderate intake of rice/pasta, and have a high intake of fast food. The prevalence of DDE was lower among children with a moderate intake of fruits (23.1%) or vegetables (22.1%). In addition, a moderate intake of vegetables had a significantly protective effect on DDE, which is consistent with previous studies (20, 21). A significant association was also found between a moderate intake of rice/pasta and an increased risk of ELS (adjusted OR, 95% CI = 1.34, 1.01–1.78), which agrees with a previous study (21). However, Cai (36) reported that fast food intake had a significantly protective effect on childhood eczema, as parents deliberately changed the original dietary habits of DDE-diagnosed children to avoid adverse risk factors for their children.

**Table 3:** Associations between dietary habits and eczema in children from southern China (*n* = 2,781).

**Category**	**Doctor-diagnosed eczema**			**Eczema-like symptoms**		
**Subcategory**	**Prevalence, n (%)**	**AOR (CI 95%)** ^**a**^	***p*** **-value**	**Prevalence, n (%)**	**AOR (CI 95%)**	***p*** **-value**
Meats intake per week			0.452^b^			0.923
Never	28.4	1.000		29.5	1.000	
1–3 times	24.1	0.760 (0.537, 1.075)		29.4	0.935 (0.680, 1.286)	
4 times or more	24.9	0.819 (0.587, 1.142)		28.5	0.937 (0.690, 1.274)	
Seafood intake per week			0.793			0.652
Never	24.2	1.000		27.8	1.000	
1–3 times	25.0	1.058 (0.874, 1.281)		29.3	1.075 (0.905, 1.278)	
4 times or more	25.9	1.071 (0.797, 1.439)		29.7	1.094 (0.837, 1.430)	
Fruits intake per week			0.112			0.710
Never	29.4	1.000		31.2	1.000	
1–3 times	23.1	0.715 (0.474, 1.080)		28.2	0.858 (0.587, 1.253)	
4 times or more	26.0	0.807 (0.536, 1.216)		29.1	0.869 (0.595, 1.268)	
Vegetables intake per week			0.195			0.820
Never	28.9	1.000		30.8	1.000	
1–3 times	22.1	0.650 (0.425, 0.995)^*^		28.4	0.867 (0.590, 1.273)	
4 times or more	25.1	0.798 (0.549, 1.161)		28.6	0.894 (0.634, 1.261)	
Beans/ grains intake per week			0.986			0.525
Never	24.8	1.000		27.3	1.000	
1–3 times	25.0	0.977 (0.793, 1.204)		29.4	1.088 (0.899, 1.315)	
4 times or more	24.8	0.954 (0.742, 1.225)		29.3	1.075 (0.858, 1.349)	
Rice/ pasta intake per week			**0.024**			**0.004**
Never	22.3	1.000		25.4	1.000	
1–3 times	27.4	1.279 (0.938, 1.745)		31.8	1.343 (1.014, 1.778)^*^	
4 times or more	23.1	1.035 (0.756, 1.416)		26.5	1.053 (0.793, 1.397)	
Eggs intake per week			0.290			0.550
Never	24.6	1.000		27.3	1.000	
1–3 times	24.0	0.943 (0.686, 1.296)		28.3	1.037 (0.779, 1.381)	
4 times or more	26.9	1.116 (0.793, 1.569)		30.1	1.138 (0.836, 1.549)	
Milk intake per week			0.149			0.381
Never	26.8	1.000		29.9	1.000	
1–3 times	23.0	0.797 (0.596, 1.066)		27.4	0.883 (0.678, 1.150)	
4 times or more	26.1	0.954 (0.717, 1.269)		29.6	0.982 (0.757, 1.273)	
Fast food intake per week			0.085			0.132
Never	25.1	1.000		28.7	1.000	
1–3 times	20.8	0.790 (0.590, 1.056)		26.5	0.917 (0.709, 1.187)	
4 times or more	35.3	1.581 (0.766, 3.262)		41.4	1.764 (0.933, 3.335)	

*Sum of the number is not 2,781 due to missing data*.

a *AOR Adjusted odds ratio, CI Confidence Interval. Adusted for Sex, Age, Birth season, Parental atopy, Breast feeding and lifetime outdoor PM_10_, SO_2_ and NO_2_*.

b *The values p < 0.05 were in bold in Pearson's Chi-square test*.

* *0.01 < p ≤ 0.05*.

**Figure 3:**
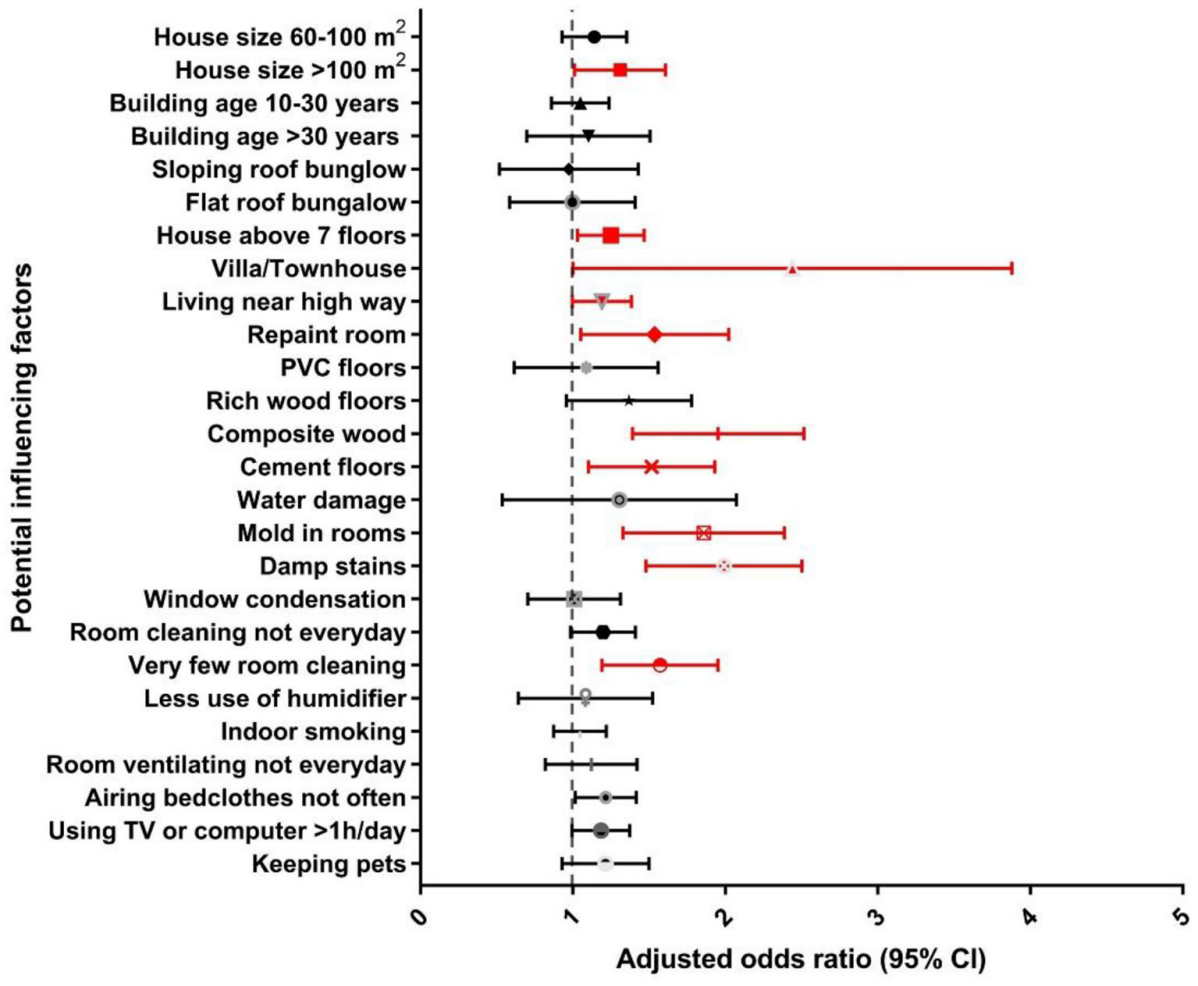
Associations between residential/household and lifestyle factors and eczema-like symptoms among children in southern China.

Figures 2, 3 presents the adjusted odds ratio for DDE and ELS associated with residential/household and lifestyle factors, respectively. Specifically, for residential factors, living in a villa/townhouse (3.10, 1.62–5.95 for DDE; 2.16, 1.16–4.00 for ELS) or in a multistory building (1.23, 1.02–1.50 for DDE, 1.24, 1.04–1.47 for ELS) with more than seven floors was significantly associated with increased risk of childhood eczema. Living near a highway was also a risk factor for childhood eczema (1.20, 1.00–1.44 for DDE; 1.18, 1.00–1.39 for ELS). Moreover, living in a larger house (>100 m^2^) had a significant positive association with increased odds of childhood eczema (1.37, 1.07–1.77 for DDE; 1.29, 1.02–1.62 for ELS).

Residential characteristics may reflect the socio-economic status (SES) of a family indirectly and determine the type and degree of outdoor pollutants, which are closely related to childhood diseases (14, 37, 38). We found childhood eczema to have significant associations with residential characteristics, including size and type of house. After lifetime outdoor PM_10_ and SO_2_ were added to the confounders, living near a highway was found to be a risk factor for childhood eczema, and the larger the house, the higher the risk of DDE in children. Living in a multistory building of more than seven floors or in a villa/townhouse was significantly associated with an increased risk of childhood eczema, which is consistent with previous studies (16, 36, 39). Our report also suggested that living near a highway is a risk factor for childhood eczema after adjusting for all of the covariates, except for NO_2_, which is consistent with previous reports (15, 39). However, several studies in Taiyuan and Shanghai, China did not find significant associations between childhood eczema and living near a highway (40, 41). Further studies on these factors are needed.

In terms of household factors, compared with stone/tile, use of composite wood and cement as floor materials was significantly associated with increasing odds of childhood eczema (Figures 2, 3). Repainting the child's room was a risk factor for ELS (1.49, 1.08–2.04). Moreover, mold or damp stains in the child's room were very significantly associated with a higher prevalence of childhood eczema.

The household environment can also produce indoor pollution, which can affect childhood diseases (14). Our report suggested that repainting the child's room was significantly associated with an increased risk of ELS, which is consistent with a Russian study (42). In particular, the risk of childhood eczema was doubled with the presence of mold or damp stains in the child's room in all models, which is consistent with previous reports (43–46). Our study also found that using composite wood, rich wood, or cement as flooring material in the child's room was significantly associated with an increased risk of childhood eczema. The same results were obtained in Shanghai, China (36). These results may have occurred because composite wood, rich wood and cement materials have greater hygroscopicity (39), which will cause invisible micro-mildew, especially in the hot and humid city of Shenzhen. Fungi in the mildew could become allergens, stimulating the body to produce specific immunoglobulin E (IgE), and eventually causing allergic dermatitis such as eczema (47).

Lifestyle factors were also found to associated with DDE and ELS (Figures 2, 3). Rare cleaning of the child's room had a significant association with an increased risk of childhood eczema in all models (Model 3: 1.51, 1.16–1.98 for DDE and 1.18, 0.99–1.42 for ELS). The frequency of airing bedclothes and keeping pets at home were also risk factors for childhood eczema. Notably, we also found that too much time watching TV/playing with a computer (> 1 h per day) was significantly related to an increased risk of childhood eczema (1.27, 1.07–1.52 for DDE and 1.17, 1.00–1.38 for ELS).

We found that childhood eczema has significant associations with lifestyle, including frequency of cleaning the child's room and airing bedclothes, keeping pets at home, and the child's time spent watching TV/playing with a computer. These results are consistent with those reported from Shanghai and Japan; not cleaning children's rooms or airing bedclothes frequently increased the risk of eczema in children (36, 48). However, we also found that there was no significant association between childhood eczema and rarely airing bedclothes. This may be because parents may change their cleaning habits when their children develop symptoms of eczema, inadvertently engaging in the protective factors that are beneficial to children's health. Another study, in Baotou city, showed that use of a humidifier increased the prevalence of childhood eczema (49). However, our results are consistent with those reported from Shanghai, with no association found between humidifiers and eczema (36). This result is probably because the climate in southern cities is always humid. Keeping pets at home was also significantly associated with an increased risk of childhood eczema, which coincided with a previous report (22). Notably, we also found another interesting result that spending too much time watching TV/playing with a computer was significantly associated with an increased risk of DDE. Despite the lack of a firm explanation for this observation, exposure time to electronic products might be inversely linked to time engaged in exercise.

The environmental pathogenic mechanisms of eczema in children may be multifaceted and have not been fully understood, which may be related to the dysregulation of cytokines and inflammatory mediators directly or indirectly induced by chronic exposure to xenobiotics (50–52). Besides, evidence have proved that indoor exposure to exogenous microbiota may alter intestinal microbiota in children, and thus regulate the immunological functions (53–55). In addition, indoor wood materials and coating on the walls may release organic pollutants such as organophosphates or brominated flame retardants, which may also exert hazardous effects on gut microbiota and potentially impair immune systems (56, 57). Nevertheless, complete skin barriers are crucial to prevent the occurrence of eczema (58), while the use of humidifiers and keeping pets at home may bring a risk to weaken the stratum corneum. We also found that eating fast food may be an influencing factor, which may be due to altered intestinal microbiota, or the intake of fatty acid and concomitant inflammation (59, 60).

The present study had some strengths. Above all, this study had a relatively large sample size, representing children in the general population. In addition, the response rate of the questionnaires was high (98%), which ensured the reliability and consistency of the results. Furthermore, the questions on atopic eczema in our study were taken from the ISAAC study (61), which was validated in previous worldwide studies (62). Nevertheless, several limitations also existed in this study. First, limited by the cross-sectional design, a cause-effect relationship could not be established. Second, information on ELS mainly came from the observations of parents, and the definition of ELS was more general in the questionnaire than in a clinical setting. Therefore, the prevalence of eczema may be overestimated. Third, the significant associations presented in this study could still be confounded by other factors. For example, some of these significant associations may disappear if the SES of the parents were included in the models. Another limitation in study design is the sensitization status of the study subjects is not considered. Besides, eczema is a strongly genetic disease with environment acting as the precipitating factor, while gene-environment interaction was not included in this study, which warrants further investigation.

## Conclusion

Our study indicated significant associations between childhood eczema and both living environment and lifestyle habits after adjusting for covariates including the child's sex, age, and birth season, parental atopy, breast feeding, and ambient air pollution, with lifetime outdoor PM_10_/SO_2_/NO_2_ values. Floor materials, repainting, house size and type, living near a highway, the frequency of cleaning the child's room and airing bedclothes, mold or damp stains in the child's room, pet-keeping at home, time spent watching TV/playing with a computer, and a moderate intake of rice/pasta were found to be risk factors for childhood eczema. A moderate intake of vegetables and fruits could decrease the risk of childhood eczema. Parents and kindergarten staff should make an attempt to avoid giving their children too many electronics-based tasks and create a low risk living environment for children during their growth stage.

## Data Availability Statement

The raw data supporting the conclusions of this article will be made available by the authors, without undue reservation.

## Ethics Statement

The studies involving human participants were reviewed and approved by the Ethics Committee of Shenzhen Institute of Information Technology. Written informed consent to participate in this study was provided by the participants' legal guardian/next of kin.

## Author Contributions

YL: investigation, data analysis, and writing. SS: investigation and writing—reviewing and editing. DZ: writing—reviewing and editing. WL: data analysis. ZD and SL: supervision. All authors contributed to the article and approved the submitted version.

## Funding

This work was supported by the Shenzhen Science and Technology Project (JCYJ20180307160045548 and JSGG20180504165551779), Research platform and projects of the education department of Guangdong province (2018GKTSCX063), and with the subject of Shenzhen Institute of Information Technology (HX-227 and SZIIT2020KJ004).

## Conflict of Interest

The authors declare that the research was conducted in the absence of any commercial or financial relationships that could be construed as a potential conflict of interest.

## Publisher's Note

All claims expressed in this article are solely those of the authors and do not necessarily represent those of their affiliated organizations, or those of the publisher, the editors and the reviewers. Any product that may be evaluated in this article, or claim that may be made by its manufacturer, is not guaranteed or endorsed by the publisher.
